# Your article needs revision? – How to improve it successfully and answer to the reviewers’ comments

**DOI:** 10.11613/BM.2021.030301

**Published:** 2021-10-15

**Authors:** Daria Pašalić, Adrijana Dorotić, Patricija Banković-Radovanović

**Affiliations:** 1 Department of Medical Chemistry, Biochemistry and Clinical Chemistry, Zagreb University School of Medicine, Zagreb, Croatia; 2 Department of Medical Laboratory Diagnostics, University Hospital “Sveti Duh”, Zagreb, Croatia; 3 Department of Transfusion Medicine, General Hospital Pula, Pula, Croatia

## Introduction

Based on the editorial policy, the *Biochemia Medica* journal seeks to enable its authors to increase the quality and recognition of their work through a review process, but also to gain relevant experiences that will be useful in the preparation of their future publications. Each author hopes that after his work enters the editorial and review procedure, it will be returned to him or her with the decision to remake the work to be acceptable for publication. Over the years of the editorial experience, we have encountered several issues that unfortunately results in re-returning the manuscript for the review or rejecting the article. Due to these additional reviews, the publishing process is delayed, and all of this complicates the editorial process.

Unfortunately, some authors skip and do not understand the importance of correct acquaintance with the Instructions for authors, which leads to extensive professional and technical reviews (*1*). However, even that would not be a problem if the authors in the next stage, when they receive instructions from reviewers and editors, meet most of the requirements and professionally and logically explain why they cannot accept a correction, if any.

At the same time, when replying to the reviewers, authors very often do not comply with Journal’s instructions, which causes delays in the editorial process. To speed up the further editorial process, it is important to mark each correction made, and then answer to the reviewers, writing exactly what and where a certain correction was made in the text.

Refusing of the reviewers’ and editor’s recommendations usually leads to the rejection of the paper and can be acceptable only if authors provide professional, scientific literature base explanation for the refusing.

Therefore, this editorial is aimed to advise authors how to make a quality revision of the article in compliance with the Journal’s peer-review policy and how authors themselves can encourage a faster decision from the Journal’s editorial board (*2*).

## General remarks and general reviewers and/or editor’s requirements

This type of suggestions usually includes some basic opinion about the article altogether. It usually implies: general opinion about the quality of the presentation in association to the good scientific publishing practice; general opinion about the composition of the subject of the manuscript and data presented; if necessary, criticism about English language and recommendation to involve English lecturer for language improvement; general suggestion to avoid of any kind of formatting the text or tables, general suggestions about decisions (reject, major revision, minor revision).

Although general considerations do not give suggestions for specific actions they are meant to point out the most prominent issues regarding the manuscript and authors should consider them carefully and thoroughly. Afterwards, authors should concentrate on specific remarks that follow in order to improve the manuscript according to the specific points. Additionally, authors should be aware that they must perform the language improvement correctly and that this is their own responsibility. This means that tools such as “spell check” and “google translator” will not provide satisfactory improvement of the language used in the manuscript.

General editors’ directions also include one unique phrase for all of the manuscripts that should be revised: “If you decide to revise the work, please submit a list of changes or a rebuttal against each point which is being raised when you submit the revised manuscript. Please make sure that all changes in the revised manuscript are highlighted in colour.” When we ask for list of changes, we expect to receive properly prepared answers to the reviewers. That means that each suggestion given by the reviewer will be exactly rewritten and full-extended explanation about the corrected text as well as the position in the revised manuscript should be provided (*e.g.* page and line). Answers such as “done”, “corrected”, “added”, “omitted” *etc.* are not acceptable and complicate the revision process, delaying the decision and resulting in the additional return of the manuscript to the authors.

Furthermore, we understand that it is up-to date to use track changes tools when corrections are made. This might be acceptable for minor revisions, but it brings difficulties to the reviewers when a major revision is requested. Reviewers are provided with the PDF format of the document and therefore cannot use the link for conversion of the word macro which converts tracked changes. One revision often is not enough and reviewers and editors should reply to the authors’ answers. For better understanding, before making their new remarks, reviewers and editors often should rewrite the authors’ comments, which is more difficult and time-consuming when authors use the tool “track changes”. This is why this Journal encourages authors to use the list of changes. Short guidelines on how to prepare the most suitable reply to reviewers and revised manuscript are presented in Table 1.

**Table 1:** Guidelines on how to successfully answer to reviewers and editors’ comments

1. Read carefully the Instructions for authors available at *Biochemia Medica* web site.	
2. Each part of the corrected text must be highlighted. Highlight the text or change the colour of the font. Avoid using the option “track changes”.	
3. In a separate document (Answer to reviewers’ comments), rewrite, answer and explain each reviewers’ comment for all of the reviews received.	
3.1.	The answers should indicate exactly what changes were introduced.
3.2.	Cite exactly the part of the text that you changed (copy/paste from the text to the answers).
3.3.	If applicable, explain how and why you performed a specific correction.
3.4.	Indicate in the answer, where exactly the correction was introduced in the text, *e.g.* section of the manuscript, paragraph in the section, a page number (if necessary).
3.5.	Answers like “done”, “corrected” “added”, “omitted”, etc. are unacceptable without exact explanation – “what”, “how” and “where in the text”.
4. For rebuttals, authors should provide professional, scientific, and literature-based explanation for the refusing to make specific correction.	
5. When the document Answers to reviewers is prepared, read once again Instructions to authors to make sure that all corrections are made in accordance with the Journal’s policy.	

## Specific remarks about the Abstract

The most abundant criticism related to the abstract part includes the structure or the composition that did not comply with the Instructions for authors. Although it is indicated in the Journal’s Instructions that only original papers should have structured abstracts, reviewers might direct authors for the same approach about other type of the manuscripts if the composition of the manuscript allows this. We as editors support this opinion.

On the other hand, the oversight that is very often unintentionally made by the author(s) is that hypothesis, aim and/or conclusion of the Abstract part does not suit with the corresponding sections in the manuscript. Abstract is quite often the reader’s first contact with the article and it is very important that Abstracts communicate all essential messages of the article.

## Specific remarks about the Introduction

The most abundant recommendation for the improvement of the Introduction concerns on missing or improvement of the hypothesis and aim of the study. Very often, hypothesis and/or aim are missing at the end of the Introduction section, which is unacceptable. Every scientific article should have a clear and unambiguous aim, which corresponds with the aim in the Abstract part and is closely related to the conclusion.

## Specific remarks about the Materials and methods

Scientific methodology dictates that Materials and methods section are written in a way that every person/scientist reading them is able to replicate the same research. Therefore, it is advisable that all relevant information concerning methodology are included in the Materials and methods section (when and where was the study performed, the instruments and methods used in the experiment with precisely listed manufacturer’s town and country in parenthesis).

## Specific remarks about the Statistical analysis

Statistical analysis is a part of Material and methods section. This is also the part of the manuscript, which brings the most difficulties in writing and revising the manuscript. Therefore, we presented it separately. We advise authors to list out all methods and explain the rationale for performing it. Each result presented in the next section must be covered by a specific and appropriate statistical method. We also recommend the authors to read and consult with articles published in *Biochemia Medica*, under the section Lessons in Biostatistics. At this moment, the section includes 35 different educational articles. Some general recommendation, can be found in articles published by Simundic in 2012 and Thiese in 2015 (*3*, *4*).

## Specific remarks about the Results

The most common correction asked for this section is avoidance of repetition of the results in two ways (*i.e.*, tables and figures). The preferred mean of presentation are tables (if applicable), because they more transparent. Therefore, authors are usually asked to omit the figures, with some reasonable exceptions. Sometimes it is noticed that some data presented in the results are not covered by the methodology. And finally, authors are asked to use SI units whenever it is applicable.

## Specific remarks about the Discussion

The authors should keep in mind that the Discussion section is not a comprehensive review of the literature. Therefore, we kindly ask the authors to focus on the comparison and discussion of their results with similar and relevant investigations, and only cite the literature that is closely in association with their subject.

Very often, references for some statements are missing. It is important that every statement in the manuscript is supported with appropriate reference, not only in the Discussion section, but throughout the manuscript.

## Specific remarks about the Tables and figures

It is usually required to simplify the format of the tables, but also to indicate all the necessary information (titles and subtitles in rows and columns, explanations of the abbreviations in the footnote) to make the tables self-explanatory. This is most helpful in all phases of revisions including final technical editing (when we accept the final version of the manuscript), which is unique for journal. Simple tables, without formatting the grids or the text, dividing or merging cells are the most acceptable. It is also very often recommended to improve the legend of tables and figures in order to provide enough information for the table to be self-explanatory.

## Specific remarks about some minor requirements

Quite often, authors have problems with some minor, but important oversights, such as abbreviations, units, *etc.* It is recommended to explain the abbreviation when it is mentioned for the first time in the manuscript and from then on use only the abbreviation. We also recommend not to use non-standard abbreviations. Furthermore, we recommend using SI units (whenever it is applicable). The list of abbreviations used in the Journal, as well the list of units is available in the Instructions for authors. Also, it is important to note that Abstract, tables and figures are observed as a separate parts of the manuscript. Due to that all abbreviations should be explained separately in those sections or written only in the expanded format.
